# Pyrocarbon Humeral Head in a Shoulder Hemiarthroplasty: Preliminary Results at 3 Years Follow-Up and Review of the Current Literature

**DOI:** 10.1155/2021/6633690

**Published:** 2021-04-14

**Authors:** Eva Campos-Pereira, Luís Henrique-Barros, Rui Claro

**Affiliations:** ^1^Department of Orthopedics, Hospital Central do Funchal, Portugal; ^2^Department of Orthopedics, Centro Hospitalar e Universitário do Porto, Portugal; ^3^Shoulder Unit, Department of Orthopedics, Centro Hospitalar e Universitário do Porto, Portugal

## Abstract

Shoulder hemiarthroplasty is a viable option in young patients with an intact rotator cuff in order to preserve the native glenoid. To avoid the dreaded and expected wear of the glenoid in very active shoulders, implants with humeral head coated with a high resistant and elastic material—pyrolytic carbon—are now an option. The authors present the first pyrocarbon coated hemishoulder arthroplasty performed at our Orthopedic Department in a patient with osteonecrosis of the humeral head. At three years of follow-up, the patient is pain free and without limitations in his daily work. The Constant score was applied pre- and postoperatively, and an improvement of 32 points was reported. Larger cohorts with long-term follow-up are required to confirm our promising results.

## 1. Introduction

Osteonecrosis of the humeral head (OHH), also known as avascular necrosis or aseptic necrosis, is an uncommon disease that results from a temporary or permanent interruption of blood supply to the humeral head [1] . The humeral head is the second most common site of nontraumatic osteonecrosis but the overall prevalence is still unknown [2, 3]. What we know has been extrapolated from research on osteonecrosis of the hip. When the humeral head collapses and the shoulder function is diminished, arthroplasty is the most reliable option [4]. Since most of the patients are younger than those with osteoarthritis (OA), it is important to choose a durable implant that can withstand the high functional demands [5, 6]. However, there is still a lack of consensus on the best therapeutic option. If on the one hand, total shoulder arthroplasty (TSA) can have a rate of glenoid loosening up to 50% at 10 years [7]; on the other hand, there is the expected glenoid cartilage erosion and subsequent revision after a shoulder hemiarthroplasty (HSA) [8]. A pyrocarbon (PyC) coated humeral head in HSA was hypothesized to overcome the limitations of HSA as it is a durable implant that generates little or no cartilage wear [6, 9, 10].

The authors present the clinical and radiologic preliminary outcomes of the first PyC coated HSA performed in our Orthopedic Department at 3 years of follow-up.

## 2. Clinical Case

**Figure 1:**
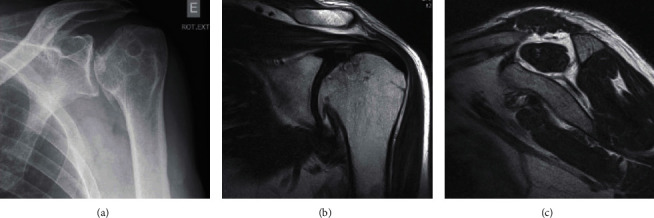
Anteroposterior plain radiograph of the left shoulder with the crescent sign, collapsed humeral head, and osteophyte (a); T2 coronal magnetic resonance image with a collapsed humeral head and large osteophyte (b); T1 magnetic resonance image with grade 1 rotator cuff muscle fatty infiltration (c).

A 48-year-old male, construction worker, with a medical history of high blood pressure, chronic obstructive pulmonary disease, diabetes mellitus, and osteonecrosis of the right femoral head was referred for shoulder consultation due to progressive left shoulder pain associated with stiffness and loss of function. On physical examination, he presented 120° of forward elevation, 10° of external rotation and internal rotation to L5, pain on passive and active mobilization, and 46 points in the Constant score. Plain radiographs and magnetic resonance imaging (MRI) of the left shoulder showed a collapse of the humeral head (stage III according to Cruess classification [11]) with a large osteophyte in the lower region and the characteristic crescent sign (Figure 1)—imaging findings consistent with OHH. Rotator cuff rupture was not objectified.

Based on age, level of activity (hard manual labour), and integrity of the rotator cuff, the patient underwent an HSA with a PyC head (Aequalis Ascend™ Flex, Tornier). The surgery was performed using a deltopectoral approach in the beach chair position. The subscapular tendon was detached using the “peeling” technique. The anatomical resection of the head was performed with the help of a cutting guide. A PyC head 48 mm × 18 mm (off-set 1.5 mm) with a 5C noncemented humeral short stem that was implanted. Intraoperatively, no acute or chronic lesions were detected in the glenoid cartilage. At the end of the procedure, a full range of motion was confirmed.

The patient followed the standard rehabilitation protocol of our service with 4 weeks of immobilization and 6 months of physiotherapy. Patient was observed for follow-up after 15 days, 6 weeks, 3 months, 1 year, and then at yearly intervals.

**Figure 2:**
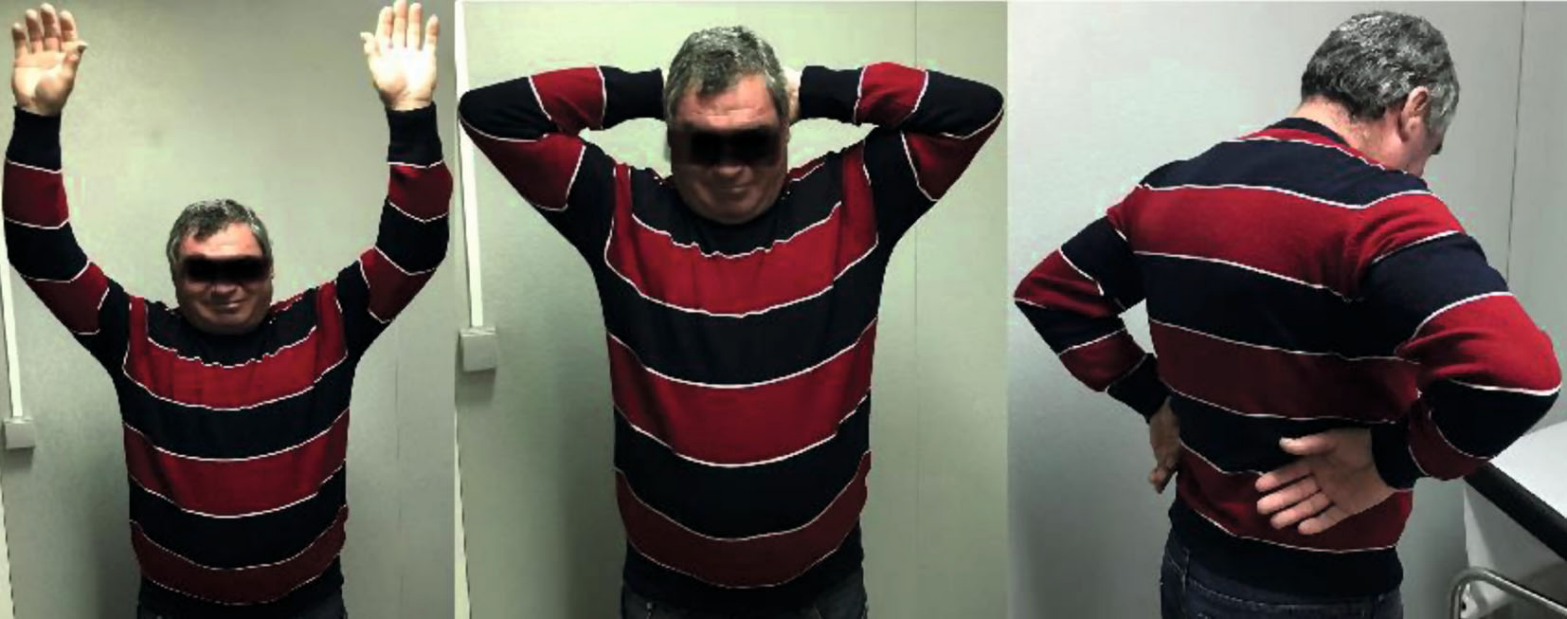
Functional outcomes three years after shoulder pyrocarbon hemiarthroplasty.

**Figure 3:**
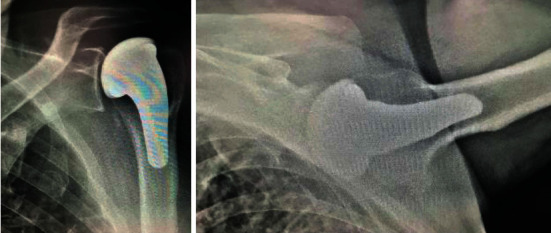
Plain radiographs of the left shoulder with good implantation of the humeral steam and no evidence of glenoid erosion.

Currently, at 3 years of follow-up, the patient remains painless, without limitations in his daily work, with 160° of forward elevation, 30° of external rotation, and internal rotation to T8 (Figure 2). The patient scored 78 points in the Constant score—an improvement of 32 points compared to the preoperative score. The radiological assessment revealed no implant failure and no glenoid erosion (Figure 3).

## 3. Discussion

Despite the imaging changes, it takes time until patients start complaining. Unlike hip joint, glenohumeral joint is neither a load joint or a constrained joint. Therefore, it can tolerate big deformities [12].

Nevertheless, when severe humeral head collapse occurs, shoulder arthroplasty, either partial or total, is an option that should be considered. Preoperatively assessment is the key to a correct diagnosis. In radiograph plains, the lesion is commonly located in the superior middle portion of the humeral head (best visualized on an anteroposterior view). With a sensitivity of almost 100%, MRI is the preferred imaging method in detecting early lesions (precollapse stages) [4]. The presence of the crescent sign represents the collapse of the subchondral bone and separation from the cartilage [4, 13].

In fact, osteonecrosis accounts for approximately 5% of all shoulder arthroplasties performed [14]. The main problem is dealing with an entity that affects younger shoulders than those with OA. The clear lack of consensus regarding the best implant in young patients is because of the limited longevity of implants in shoulders with high functional demands [7]. In 2004, Parson et al. [15] described a narrowing of 68% of the glenohumeral joint space during the follow-up interval of 43 months of HSA. Herschel et al. [8] found severe glenoid erosion in one-third of the HSA after a mean postoperative time of 2.5 years. Despite high rates of symptomatic glenoid erosion due to friction with a metallic surface (up to 21% after 5 years follow-up ^7^) and the drop of satisfaction rates over the time (satisfaction of only 25% after an average of 17 years ^5^), HSA has been accepted by most authors in young patients [6]. The good results may partially be due to a simple procedure when compared to TSA and the less difficulty achieving range of motion postoperatively [16]. Additionally, a glenoid without subchondral changes or cysts, covered by an intact rotator cuff, has favorable conditions to resist to erosion, as supported by Herschel et al. [8], and the attempt to preserve bone can be helpful in later revisions. Rispoli et al. [17] revised ten of fifty-one HSA (performed for the treatment of glenohumeral OA), which was done to treat painful glenoid arthrosis in nine of the ten. In a systematic review of all TSA and HSA for the treatment of glenohumeral OA with a minimal follow-up of 7 years, Bekerom et al. [18] identified a higher revision rate for any reason in the HSA group (13%) than the TSA group (7%). Therefore, when the expected cartilage wear becomes painful, it is recommended to perform the revision of the HSA [15–17].

In contrast, because of the unreliable outcomes of HSA and the uncertain about the wear predisposing factors, some authors prefer TSA as the primary treatment of OHH. Some refer better outcomes in short and middle terms [6]. Sperling et al. [19] concluded that TSA seems to be the preferred procedure for pain relief, improvement in abduction, and to lowering the risk of revision surgery, among those with an intact rotator cuff. Nevertheless, the durability of the glenoid component is limited and becomes symptomatic or fails at 1.2% per year [5]. The glenoid loosening accounts for 24% of all TSA complication [20]. This weak spot is also well demonstrated in the study of Feeley et al. [21] that reported complication rates of 22% and 8% in TSA and HA, respectively, in the treatment of OHH and in the study of Gonzalez et al. [22], with the same complication rates following TSA.

To overcome the main trigger for revision in HSA—symptomatic glenoid—PyC coated HSA was developed [8, 9, 16]. This manmade biomaterial has the best combination of blood compatibility and physical and mechanical properties [9]. PyC has demonstrated decreased bone wear compared to metal in in vitro models [10], and it is believed to promote neosynthesis of a cartilage-like tissue [7]. Despite previous orthopedic applications in small arthritic joint such as the hand and wrist, outcomes in the glenohumeral joint are beginning to emerge. Yet, most of the studies report outcomes in interposition arthroplasty—pyrocarbon coated interposition shoulder arthroplasty (PISA)—and some even compare to HSA and TSA [5–7, 9]. The lack of results of PyC coated HSA is notorious. In 2018, Klawitter et al. [10] supported the use of PyC in humeral head HSA instead of the conventional metal HSA and demonstrated less damage to the bone in simulated tests for PyC HSA when compared to cobalt chromium implants in in vitro models. A recent prospective multicenter study performed by Garret et el [6]., that included 65 patients who underwent PyC HSA, demonstrated improvement in pain and function in patients with primary and secondary OA.

We report preliminary outcomes of the first PyC coated HSA performed in our Orthopedic Department, with a follow-up of 3 years, with very encouraging results. The patient is able to perform all ranges of motion, and the radiographic assessment demonstrates good implantation of the humeral stem and no evidence of secondary OA of the glenoid. Our results—improvement in the Constant score and unchanged glenoid after 3 years of follow-up—were similar to those reported in the atraumatic OHH group assessed in one of the largest prospective multicenter study enrolling PyC HSA [6]. Therefore, satisfactory functional recovery in a young and active patient without uneventful events turns this treatment into an attractive option in patients with OHH. Yet, further investigations with larger cohorts assessing the radiographs and functional outcomes are necessary to confirm the safety and performance of PyC HSA in a long-term follow-up.

## Data Availability

No data are available.
